# Stress CMR myocardial perfusion imaging (CMR-MPI) is cost-effective compared to nuclear SPECT: a retrospective cost-effectiveness analysis

**DOI:** 10.1186/1532-429X-14-S1-O3

**Published:** 2012-02-01

**Authors:** Sanjeev Francis, Joshua Cohen, Natalia Olchanski, Otavio R Coelho-Filho, Bobby Heydari, Ravi Shah, Marcia B Leavitt, Henry Gewirtz, Raymond Kwong

**Affiliations:** 1 Cardiology, Massachusetts General Hospital, Boston, MA, USA; 2 Cardiology, Brigham and Women's Hospital, Boston, MA, USA; 3 Tufts Medical Center, Boston, MA, USA

## Background

Stress CMR myocardial perfusion is a strong risk-stratifying tool increasingly used for patient management. However, the cost-effectiveness of this technique in patients with suspected ischemia, has never been studied against nuclear SPECT.

## Methods

From 2003-2011, 707 patients underwent CMR-MPI for ischemia assessment in our center. Estimated pre-test cardiac risk derived from a combined Framingham Heart Study and Diamond Forrester risk percentage was used to match CMR patients against 39,876 patients who underwent pharmacologic stress SPECT in another tertiary-care center during the same time period. Framingham scoring system for the prediction of cardiovascular risk was also stratified by presence or absence of prior evidence of CAD. A validated computer algorithm was used to perform 1:1 patient risk-matching. For all patients, cardiac events (acute MI/death), angiographically-significant CAD, percutaneous coronary intervention and bypass grafting, repeat stress testing/imaging within 2 years, and cost estimates for these events from national average were collected for cost-effectiveness analysis.

## Results

One-to-one risk-profile matching between CMR-MPI and SPECT was successful in 704 patients (99.6%). Ischemia by SPECT was positive, negative, and equivocal in 8%, 74%, and 18%, which compared with 22%, 75%, and 3%, respectively by CMR-MPI. A negative SPECT was associated with a 2-year cardiac event rate of 4.6% compared to 1.3% by CMR-MPI (p=0.002). Other items relevant to cost-effectiveness analysis using imaging for “gate-keeping” stratification are shown in Table 1.

**Table 1:** 

	CMR-MPI Negative or equivocal for Ischemia	SPECT Negative or equivocal for Ischemia
Cardiac Event Rate (%/2 years)	N=9 (1.3%)	N=32 (4.6%)
Need for Repeat Imaging within 2 years	N=27 (6%)	N=99 (14%)
Need for coronary angiography within 2 years	N=24 (3%)	N=155 (22%)
PCI or Bypass Surgery Performed within 2 years	N=15	N=46
Estimated Cost of SPECT or CMR-MPI (N=705)	$704,000	$598,400
Estimated Costs of Related Patient Care over 2-yrs ($)	$789,642	$3,419,355

## Conclusions

In patients with an intermediate risk of ischemic heart disease, stress CMR myocardial perfusion is cost-effective when compared to pharmacologic stress SPECT. A negative stress CMR perfusion study is associated with a lower 2 year event rate and lower downstream costs.

## Funding

Dr. Kwong is in part supported by the National Institutes of Health R01HL091157. All other authors have reported that they have no relationships relevant to the contents of this paper to disclose.

